# Amorphous and Co-Amorphous Olanzapine Stability in Formulations Intended for Wet Granulation and Pelletization

**DOI:** 10.3390/ijms231810234

**Published:** 2022-09-06

**Authors:** Nuno F. da Costa, Rolf Daniels, Ana I. Fernandes, João F. Pinto

**Affiliations:** 1 iMed.ULisboa—Research Institute for Medicines, Faculdade de Farmácia, Universidade de Lisboa, Av. Prof. Gama Pinto, 1649-003 Lisboa, Portugal; 2 Department of Pharmaceutical Technology, Eberhard Karls University, Auf der Morgenstelle 8, D-72076 Tuebingen, Germany; 3 CiiEM—Interdisciplinary Research Center Egas Moniz, Instituto Universitário Egas Moniz, Monte de Caparica, 2829-511 Caparica, Portugal

**Keywords:** (co-)amorphous, dissolution, granule, olanzapine, pellets, stability, sulfonic acid

## Abstract

The preparation of amorphous and co-amorphous systems (CAMs) effectively addresses the solubility and bioavailability issues of poorly water-soluble chemical entities. However, stress conditions imposed during common pharmaceutical processing (e.g., tableting) may cause the recrystallization of the systems, warranting close stability monitoring throughout production. This work aimed at assessing the water and heat stability of amorphous olanzapine (OLZ) and OLZ-CAMs when subject to wet granulation and pelletization. Starting materials and products were characterized using calorimetry, diffractometry and spectroscopy, and their performance behavior was evaluated by dissolution testing. The results indicated that amorphous OLZ was reconverted back to a crystalline state after exposure to water and heat; conversely, OLZ-CAMs stabilized with saccharin (SAC), a sulfonic acid, did not show any significant loss of the amorphous content, confirming the higher stability of OLZ in the CAM. Besides resistance under the processing conditions of the dosage forms considered, OLZ-CAMs presented a higher solubility and dissolution rate than the respective crystalline counterpart. Furthermore, in situ co-amorphization of OLZ and SAC during granule production with high fractions of water unveils the possibility of reducing production steps and associated costs.

## 1. Introduction

High throughput screening is a simple and quick procedure used to test a large set of molecules with potential biological activity [1] and, thus, accelerate the discovery of novel drug substances [2]. Unfortunately, the synthesis of rather complex chemical entities is leading to the ever-increasing number of hydrophobic drugs with poor solubility in water [3,4,5,6]. These drugs, when processed into dosage forms (e.g., granules, pellets or tablets), fail to dissolve and, consequently, present low bioavailability and do not reach the plasmatic concentration required to trigger the desired therapeutic effect [7,8].

Crystal engineering alters the intermolecular interactions responsible for holding the molecules together, affects the packing of the molecules and impacts the physical properties of solids (e.g., compressibility, solubility and dissolution rate) [9,10,11]. In this respect, the disarrangement of the crystalline structure of drugs by the production of their amorphous counterparts is regarded as one of the most promising strategies to enhance drug solubility and bioavailability [12,13,14]. In fact, amorphous materials, unlike their corresponding crystalline materials, possess high free energy, i.e., low enthalpy and high entropy [15], which positively affects properties such as solubility in water and dissolution rate [12,16]. Unfortunately, the disorder of the molecular arrays also negatively affects the stability of the amorphous materials (e.g., due to chemical instability and higher hygroscopicity), which are prone to recrystallization towards the most stable crystalline counterpart, thus failing to enhance bioavailability as expected [12].

Co-amorphous systems (CAMs), i.e., dispersions at the molecular level of drugs in low molecular weight compounds, have gained special interest [17,18,19,20,21] by enhancing the stability of the amorphous systems produced while increasing the solubility of the drugs [20,22]. The combination of drugs with a second substance (either a pharmaceutically accepted excipient or a second drug providing a sensible therapeutic combination [17,23,24,25]) enables the stabilization of the amorphous system, due to the establishment of intermolecular bonds between the two entities, often with a higher glass transition temperature (T_g_) than the pure amorphous drug(s) or the physical mixture of the individual components [23,26]. Additionally, ternary systems have also been recently considered to ameliorate the stability of CAMs by dramatically increasing T_g_ [27]. Since amorphous, binary and ternary CAMs are intrinsically unstable [28,29], the evaluation of recrystallization, namely due to stress manufacturing conditions (e.g., heat, moisture or pressure), is of paramount importance.

Olanzapine (OLZ), 2-methyl-4-(4-methyl-1-piperazinyl)-10*H*-thieno-[2,3-b][1,5]benzodiazepine, is one of the most common antipsychotic drugs on the market [30], which is frequently used in the treatment of schizophrenia, bipolar disorder and other related psychoses [31]. Although there are interesting therapeutic advantages offered by OLZ [32,33], its poor water solubility and bioavailability [32] reduce the therapeutic effectiveness of the treatment. In fact, as a drug belonging to class II of the biopharmaceutical classification system (BCS) with many transient polymorphs (e.g., the most stable form I polymorph was considered in this study, as a control), it benefits from a transformation at the molecular level to overcome such limitations. Previous studies have shown that the preparation of amorphous OLZ by quench cooling could be used to enhance the solubility of the drug (3.5-fold enhancement compared to crystalline OLZ, 141.4 ± 1.5 vs. 40.6 ± 1.4 mg/L, in phosphate buffer pH 8.0) [34]. However, the low stability of the system resulted in the rapid recrystallization of the amorphous drug back to its crystalline counterpart. To increase the stability of amorphous OLZ, co-amorphization of the drug with saccharin (SAC) has been attempted [35]. Unfortunately, the co-amorphization of OLZ resulted in a cohesive and non-free-flowing powder, impairing its handling per se (tableting or capsule filling) into dosage forms [36,37] and requiring the use of further down-streaming processing to overcome these drawbacks. 

The manufacture of granules, by wet granulation, and pellets, by extrusion and spheronization, has been extensively considered to overcome the poor flowability of solids, improve the compressibility of materials or control the release of drugs from the dosage forms [38,39,40]. The manufacture of granules or pellets involves, firstly, the addition of a granulation liquid to a blend of powdered materials to promote agglomeration into granules or to allow extrusion and spheronization, before drying of the formed agglomerates [39,41]. Thus, the combined effect of water, shear forces and temperature used, raises questions on the stability of amorphous and CAM systems regarding the probable recrystallization of drugs [17,42,43]. This work aimed to assess the stability of OLZ-amorphous and OLZ-CAM when subjected to different stress conditions (e.g., water and heat) required for their processing into granules, extrudates or pellets, which may be the final dosage forms, or further converted into tablets or capsules, before patient administration.

## 2. Results and Discussion

### 2.1. Preparation of Partially Amorphous and Co-Amorphous Olanzapine

Co-amorphization of OLZ, using SAC as co-former, has been previously reported to enhance the solubility (approximately 145 and 40-fold increase, respectively, as compared to the solubility of crystalline and amorphous OLZ) and stability of the drug (OLZ-CAM > 24 weeks vs. amorphous OLZ < 1 week at 75% RH/25 °C) [34,44]. In the present work, amorphization of the pure drug by quench cooling or co-amorphization with a co-former (SAC) by solvent evaporation was confirmed using X-ray powder diffraction (XRPD) and differential scanning calorimetry (DSC) characterization methods. 

**Figure 1:**
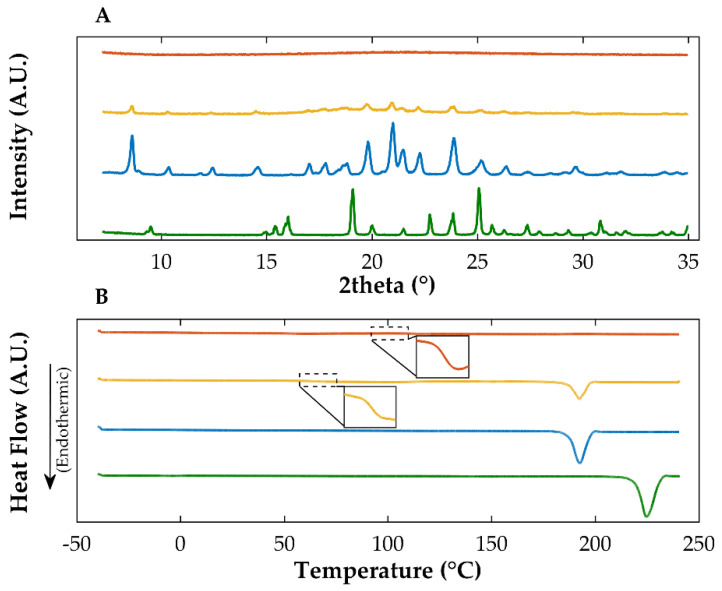
X-ray diffractograms (**A**) and thermograms (**B**) of co-amorphous olanzapine: saccharin (orange), amorphous olanzapine (yellow), crystalline olanzapine (blue) and crystalline saccharin (green). The inserts in thermogram (**B**) are magnifications of the glass transitions observed in the amorphous and co-amorphous entities.

Diffractograms of quench cooled OLZ showed a halo pattern (Figure 1A), which suggests the amorphization of the drug. However, residual traces of crystallinity were detected on the diffractograms, as indicated by the presence of low intensity diffraction peaks, characteristic of crystalline OLZ. A T_g_ (at 67 °C), was observed in the thermograms attributable to the amorphous form of the drug (Figure 1B). However, recrystallization of the amorphous drug was detected at 103.3 °C, followed by a melting event at 192.5 °C, consistent with the polymorphic form I of OLZ. The combination of data collected from the calorimetric and the diffractometric studies has strongly suggested that OLZ was mostly in the amorphous state [35].

For the sample obtained from solvent evaporation, the diffractometry data have shown the existence of a halo pattern and the absence of characteristic diffraction peaks related to the crystalline form of OLZ and SAC, suggesting the complete amorphization of both compounds when in combination (Figure 1A). Accordingly, thermograms have shown a unique T_g_ at 100.8 °C and the absence of melting events that could have been related to either compound, suggesting good miscibility between them (Figure 1B).

### 2.2. Preparation of Granules, Extrudates and Pellets Containing Crystalline, Partially Amorphous and Co-Amorphous Olanzapine

During the production of solid dosage forms, the occurrence of solid-state conversions of drugs is well described in the literature [45,46,47] and can seriously impact the solubility and dissolution rate of drugs [35,45]. Therefore, it is paramount to understand how the processing of crystalline, amorphous and OLZ-CAM, used as a model drug, may promote changes in the molecular arrangement of the drug. 

#### 2.2.1. Diffractometric and Calorimetric Characterization of Materials

The stability of crystalline OLZ (formulation A) in granules, extrudates and pellets was confirmed using calorimetry (DSC) and diffractometry (XRPD). Processed samples presented diffraction peaks at the same position, compared to the pure crystalline OLZ and the physical mixture (formulation A), and intensity, compared to the physical mixture, suggesting the maintenance of the crystallinity of the drug (anhydrous polymorphic form I) (Figure S1—Supplementary Materials). Similarly, thermograms of samples obtained after processing confirmed the presence of the crystalline polymorphic form I of the drug as shown by the presence of a single melting event (correspondent to the melting of OLZ—192.4 °C [35]) at the same temperature and with similar enthalpies compared to those observed in samples taken from the physical mixture containing the crystalline drug (192.4 ± 0.3 °C and 35.4 ± 2.8 J/g, respectively) (Figure S1). Thus, it can be suggested that crystalline OLZ did not undergo modifications in its crystalline arrangement during the processing into solid dosage forms, regardless of the fraction of water used to wet the materials and granulate the powders, or the drying temperature applied.

In contrast, the poor physical stability of amorphous materials may determine the recrystallization of the amorphous form back into its crystalline counterpart [12]. Both water and heat used in the manufacturing process can be considered stress conditions to amorphous systems, likely to promote recrystallization [48].

**Figure 2:**
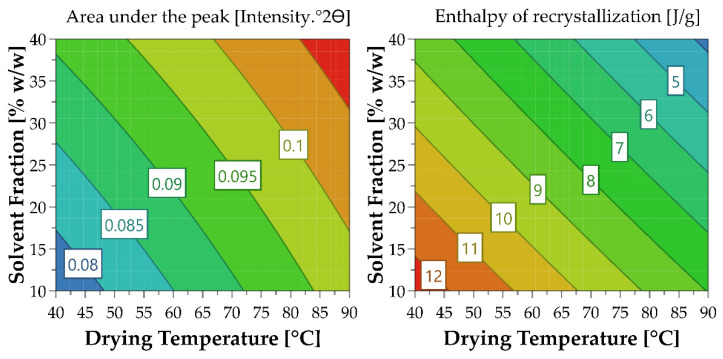
Effect of the drying temperature and solvent fraction (% *w*/*w*) on the extent of OLZ recrystallization, as determined by X-ray powder diffraction (area under the peak of OLZ, 20.7–21.2 °2θ) and differential scanning calorimetry (enthalpy of recrystallization).

In this work, throughout the manufacture of granules, extrudates or pellets, different fractions of water were used as granulation liquid, and drying temperatures were imposed on mixtures containing the amorphous drug. In fact, the application of these stress conditions led to a significant increase in the degree of recrystallization of the amorphous OLZ, as suggested by the analysis of the diffractograms and thermograms (Figure 2). Previous studies from our group have shown that the area under the most intense diffraction peak of OLZ (between 20.7 and 21.2 °2θ) correlates well with the fraction of amorphous OLZ and may be used to predict the degree of crystallinity of samples [35]. The physical mixtures containing crystalline or amorphous OLZ presented areas under the respective diffraction peaks of 0.107 ± 0.003 or 0.079 ± 0.002 intensity.°2θ, respectively (Table 1). In a similar fashion, the analysis of DSC thermograms allowed the measurement of the enthalpy of recrystallization, which was used to infer the recrystallization of amorphous OLZ in samples. The physical mixture containing amorphous OLZ presented an enthalpy of recrystallization of 12.61 ± 0.11 J/g, while thermograms of the physical mixture containing crystalline OLZ have not shown the recrystallization of the drug, due to the absence of amorphous content in the samples (Table 1). 

**Figure 3:**
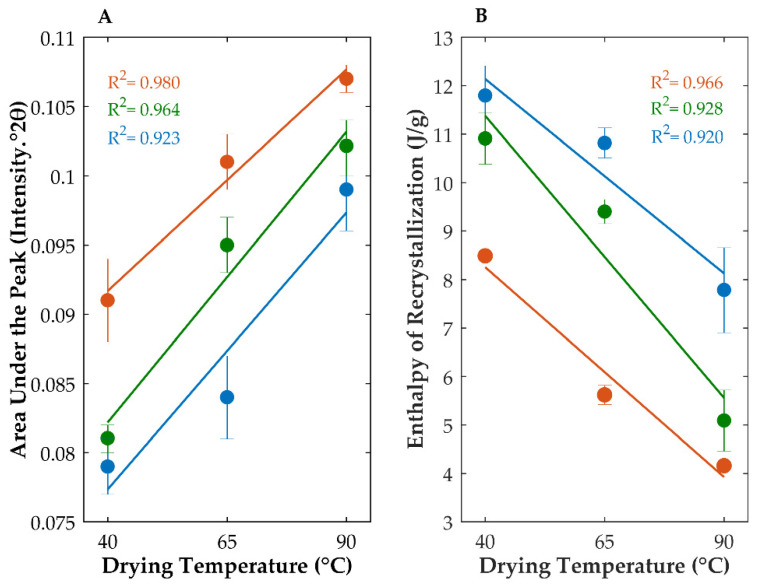
Influence of the drying temperature on the extent of recrystallization of amorphous olanzapine when 10% (blue), 25% (green) or 40% (orange) of water were applied to mixtures, as determined by X-ray powder diffraction (**A**) and differential scanning calorimetry (**B**).

Both characterization techniques revealed that the recrystallization of amorphous OLZ was related to the fractions of water used in the processing. In fact, diffractograms of samples granulated with the highest water fraction presented the highest areas under the OLZ characteristic peaks (0.079 ± 0.002, 0.081 ± 0.001 and 0.091 ± 0.003 intensity.°2θ, for samples granulated with 10, 25 or 40% of water, respectively, and dried at 40 °C) and reduced enthalpies of recrystallization (11.80 ± 0.61, 10.91 ± 0.53 and 8.49 ± 0.09 J/g for the same samples (Figure 2, Table 1)). As for the temperature used to dry the granules, unsurprisingly the highest temperature (90 °C) promoted the highest extent of recrystallization, irrespective of the fraction of water applied to granulate the powdered materials [0.107 ± 0.001 intensity.°2θ (diffraction) and 4.16 ± 0.10 J/g (enthalpy of recrystallization) for samples granulated with 40% of water and dried at 90 °C) (Figure 2). This high extent of recrystallization may be explained based on the fact that this temperature was well above the T_g_ of the amorphous OLZ (rubbery state) with the respective molecules presenting high mobility, which allowed their rearrangement into a more stable physical form. Traces of amorphous OLZ were still present in the samples after granulation and drying with high fractions of water and heat, based on data related to the quantification of the enthalpy. One can hypothesize that the short time required to dry the granules at 90 °C was insufficient to promote the total recrystallization of the drug. Furthermore, the recrystallization of amorphous OLZ at 40 or 65 °C may be explained by a combined effect with water, based on the plasticizing effect of water molecules, which may have promoted a decrease in the T_g_ of amorphous materials favoring the arrangement of molecules [48]. To gain a better insight into the impact of the drying temperature and solvent fraction on the extent of recrystallization of amorphous OLZ, the results of the area under the peak and the enthalpy of recrystallization were modelled. These models presented an R^2^ above 0.940, confirming the fitting of the models to the data. The contour plot, in Figure 2, clearly shows the positive effect of both drying temperature and solvent fraction on the extent of recrystallization of the drug. Interestingly, the extent of recrystallization of amorphous OLZ has shown a linear correlation (R^2^ > 0.920) with the temperature required to dry the granules (Figure 3).

It is worth mentioning that the diffraction peaks observed upon recrystallization of OLZ were consistent with those observed for the most stable polymorphic form of the raw drug material (form I) indicating that recrystallization of the amorphous OLZ was accomplished by the growth on the pre-existing crystalline seeds of the form I of OLZ in the quench cooled drug. 

Additionally, recrystallization of OLZ was drastically promoted when masses containing 40% of water were stored for 24 h (Table 1). According to previous studies [49,50], high humidity conditions may lead to the disruption of the interactions established between molecules, with a negative impact on the stability of the amorphous system, in line with the present results. The rupture of intermolecular interactions within the amorphous structure of OLZ allowed the rearrangement of the more mobile molecules into crystals. Similarly, the shear force applied to the wet mass while extruding promoted partial recrystallization of OLZ. As a result, extrudates and pellets containing OLZ predominantly in the crystalline form were prepared. Thus, the results obtained not only highlighted the instability of amorphous OLZ throughout the process of manufacture of dosage forms, particularly, extrudates and pellets, but also emphasized the effect of different times of exposure to stress conditions. 

**Figure 4:**
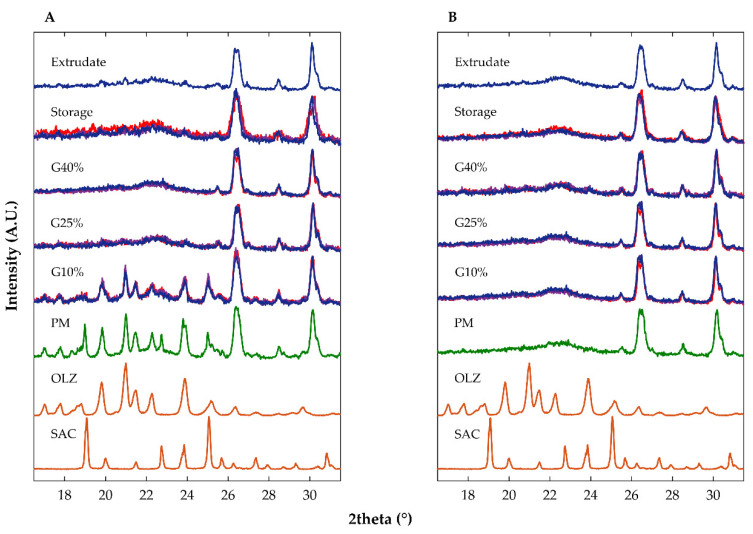
Diffractograms of pure crystalline saccharin (SAC), pure crystalline olanzapine (OLZ) and samples containing olanzapine and saccharin (formulation B) as crystalline forms (**A**) and co-amorphous forms (**B**) after physical mixing (PM), wet granulation (G, using 10, 25 or 40% of water), storage (10% of water) and extrusion. Drying temperature applied to samples: 40 °C (dark blue), 65 °C (purple) and 90 °C (red).

Diffractograms of the physical mixture containing OLZ, SAC and the other excipients (Table 2, formulation B) presented intense diffraction peaks at 19.0, 19.9, 21.0, 22.3, 23.8 and 25.0 °2θ (Figure 4). Samples from granules made with 10% of water presented diffraction peaks superimposable to those of the physical mixture, regardless of the drying temperature considered, suggesting that no solid-state modifications in the crystalline arrangement of the drug occurred (Figure 4). Noteworthy is that the physical mixture containing OLZ and SAC, granulated 24 h after production with 10% of water, yielded samples where co-amorphization of OLZ and SAC seems to have occurred, as ascertained by diffractograms (Figure 4). The incorporation of either 25 or 40% of water in mixtures resulted in the preparation of a viscous and sticky mass which made further processing of the materials difficult. Similar difficulties were observed when OLZ-CAM was used as a feedstock material. Interestingly, the drying of these wet masses, regardless of the temperature used, resulted in the production of samples with a significant reduction in the intensity of the diffraction peaks of both crystalline OLZ and SAC and presented the halo pattern that is characteristic of amorphous materials. 

To understand the viscous and sticky behavior of masses, a placebo control blend (without OLZ) containing SAC at a fraction of 18% (formulation B, 57% of dibasic calcium phosphate anhydrous) was prepared. The incorporation of 25 or 40% of water in the placebo control sample resulted in the preparation of free-flowing granules. The formation of the viscous mass may therefore be related to the joint presence of OLZ and SAC in the mixture.

**Figure 5:**
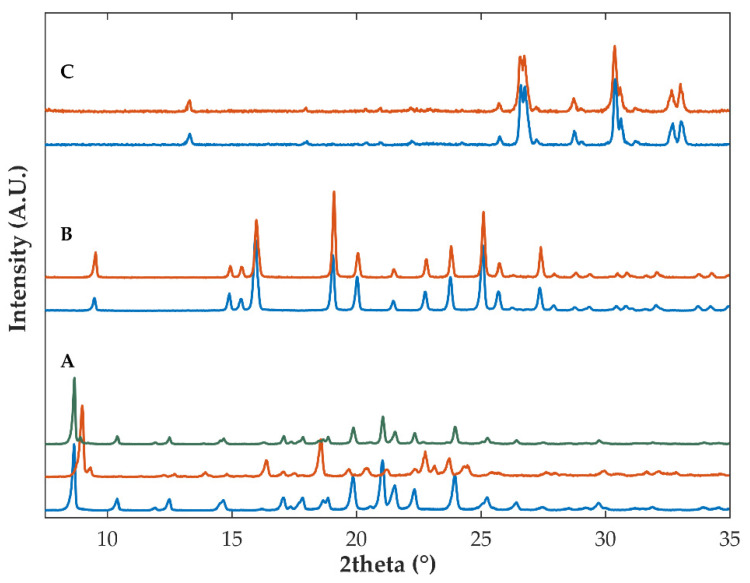
Diffractograms of control samples containing either olanzapine (**A**), saccharin (**B**) or the placebo-based formulation (**C**) before (in blue) and after (in orange) exposure to 25% of water. The diffractogram of olanzapine in green represents the sample containing wetted olanzapine after drying under room conditions (23 °C/65% RH).

To support the in situ co-amorphization of OLZ and SAC, samples containing pure crystalline OLZ, pure crystalline SAC or the placebo-based formulation were wetted with 25% of water. After exposure to water, samples were analyzed using diffractometry and spectroscopy (Fourier-transformed mid infrared spectroscopy, FTIR). Diffractograms of the SAC or the placebo-based samples, pre-wetted with 25% of water, were equivalent to those of un-wetted samples (Figure 5), suggesting the maintenance of the solid-state arrangement of samples during exposure to water. 

Inversely, diffractograms of samples made of OLZ and water presented noticeable modifications by comparison with those of un-wetted OLZ (anhydrous polymorphic form I). From the comparison of the diffractograms obtained with those available in the literature, it was concluded that the wetting of OLZ with 25% of water resulted in the production of the hydrated form of OLZ (di-hydrate B) (Figure 5) [31,51]. FTIR spectra confirmed the preparation of the hydrated form of OLZ and suggested the disruption of the intermolecular bonds established in the process, between the dimers of OLZ. Consequently, water seems to have induced rupture of intermolecular bonds and favored the interaction between OLZ and SAC, probably forming a salt due to the marked difference in pKa of the entities (ΔpKa > 3 units [52]). Noteworthy is that, after drying under room conditions (23 °C/65% RH), the control sample containing OLZ and water presented diffraction peaks aligned with those of the polymorphic form I of the drug (Figure 5). These results are supported by the studies conducted by Petry et al., who have observed the in situ co-amorphization of indomethacin and arginine when masses were exposed to high humidity conditions (23 °C and 75% RH) [53]. 

Therefore, the study suggested that the impregnation of dried blends with water or a long exposure time of the powdered blend to water induced the formation of the OLZ-CAM. The in situ amorphization of OLZ during the process of granulation offers inherent advantages compared to the commonly used processes of amorphization of drugs (e.g., quench cooling or solvent evaporation), such as a reduction in processing costs and time. Furthermore, the concerns related to the stability of amorphous and CAM materials are expected to be circumvented, as described elsewhere [54,55,56,57]. Petry et al. [55] observed that the coat of tablets containing a mixture of crystalline indomethacin and arginine resulted in a change of coloration of the compacts from white to yellow, considered an indicator of the amorphization of the drug [56,58]. Similarly, the same change of coloration was observed when tablets were stored at 75% RH at room temperature for 26 days, regardless of the presence of other excipients in the coat. To further support the in situ amorphization of indomethacin, samples were characterized using XRPD and FTIR and the data supported the preparation of the indomethacin:arginine CAM. In another study, Marsac et al. [57] prepared compacts made of binary mixtures of poly(ethylene oxide) and ibuprofen, ketoprofen, acetaminophen, naproxen, felodipine or ketoconazole. Compacts exposed to high relative humidity conditions showed a spontaneous loss of crystallinity of both the drug and polymer, which was justified by the dissolution of the crystalline drugs into the disordered regions of the polymer. Inversely, no loss of crystallinity was observed when compacts were stored in the absence of water or when the polymer was not included in formulations.

Interestingly, in the present study, when SAC was included in the formulation, extrudates could be produced if a minimum of 10% of water was added to the mixtures. Lower fractions of water (5%) hindered the extrusion of the wet masses, likely due to the high force required to obtain extrudates (>50 kN). Here, it may be assumed that OLZ-CAM presented a rubber-like flow, increasing the plasticity of the formulations and thus facilitating the extrusion of the powder mass through the die. Unfortunately, the high cohesiveness of extrudates containing OLZ and SAC prevented the production of pellets. The cohesiveness of OLZ-CAM was described previously [36,37]. 

As previously mentioned, the incorporation of the OLZ-CAM as starting material in the mixture (formulation B, Table 2) was considered to enhance the solubility in water, the dissolution rate and stability of the model drug. Figure 4 presents the XRPD diffractograms of the OLZ-CAM (physical mixture, Table 1) prior to and after the manufacture of granules and extrudates. Regardless of the processing conditions imposed on the materials, data suggested the maintenance of the amorphous form of the drug after processing, based on the absence of diffraction peaks related either to OLZ or SAC. This observation supports the viability of manufacturing solid dosage forms containing OLZ-CAM on an industrial scale. 

#### 2.2.2. Fourier-Transformed Mid Infrared (FTIR) and Near Infrared (NIR) Spectroscopy

FTIR and NIR spectroscopies are common techniques used to confirm the establishment of interactions (bonds) between molecules, as those expected to be formed during the conversion of the crystalline into the amorphous arrangement of powders, or vice versa [23,26]. 

Pure crystalline OLZ presents characteristic FTIR peaks at 3218 and 1583 cm^−1^ (N-H bonds), 2931 and 2791 cm^−1^ (C-H stretching) and 1289 cm^−1^ (C-N stretching) (Figure S2, Supplementary Materials). Likewise, NIR spectra of pure crystalline OLZ showed characteristic peaks at 5974 cm^−1^ (C-H overtone), 4651 cm^−1^ (N-H combination) and below 4450 cm^−1^ (C-H combination) (Figure S2). These peaks were observed in the spectra of samples collected from the physical mixture containing the crystalline OLZ (formulation A, Table 2), with a lower intensity due to the dilution effect of the drug in the powder blend (Figure S2). Samples of granules, extrudates and pellets manufactured with crystalline OLZ (formulation A) presented spectra identical to the ones obtained from the physical mixture prior to its processing (Figure S3, Supplementary Materials), confirming that no modifications to the drug’s crystalline network occurred, in line with the previous observations regarding the maintenance of the crystalline arrangement of the drug during the production of granules, extrudates and pellets. 

Spectral data of physical mixtures and processed blends containing OLZ-CAM were also comparable (Figure S3), indicating that the interactions established between OLZ and SAC in the CAM were preserved throughout processing, without drug recrystallization. If recrystallization of OLZ and SAC had occurred, modifications in the spectra of samples were expected, possibly leading to phase separation and a higher rate of recrystallization [26].

**Figure 6:**
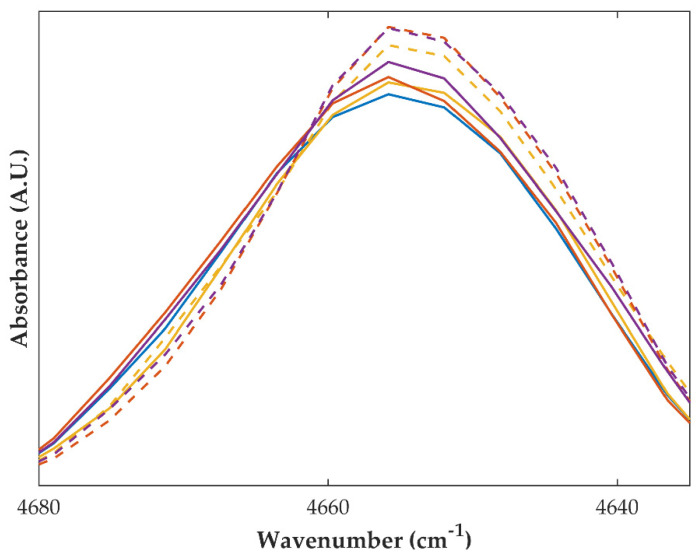
Fourier-transformed near infrared spectra of partially amorphous olanzapine in a physical mixture (blue) and granules (10%, yellow, 25%, orange, or 40%, purple, of water for granulation) dried at 40 °C (solid line) and 90 °C (dashed line).

Samples of granules, extrudates and pellets produced with partially amorphous OLZ as a starting material presented FTIR and NIR spectra with slight changes in comparison with those of the physical mixtures used as controls. Particularly, NIR spectroscopy revealed that blends exposed to higher fractions of water and higher temperatures (thus having a higher extent of recrystallization, as suggested by XRPD and DSC), presented a peak between 4680 and 4630 cm^−1^ (associated with the N-H bonds) with higher intensity than the samples presenting a lower extent of recrystallization because the powder blend was not so much exposed to the stress conditions (lower water and heat) (Figure 6). The polymorphic crystalline form I of OLZ is organized through N-H hydrogen bonds [31], and therefore, an increase in the intensity of the respective peaks foresees a higher number of bonds established between the molecules of OLZ, reflecting the degree of recrystallization of the amorphous OLZ and reinforcing the previous observations regarding the recrystallization of amorphous OLZ (Table 1).

**Figure 7:**
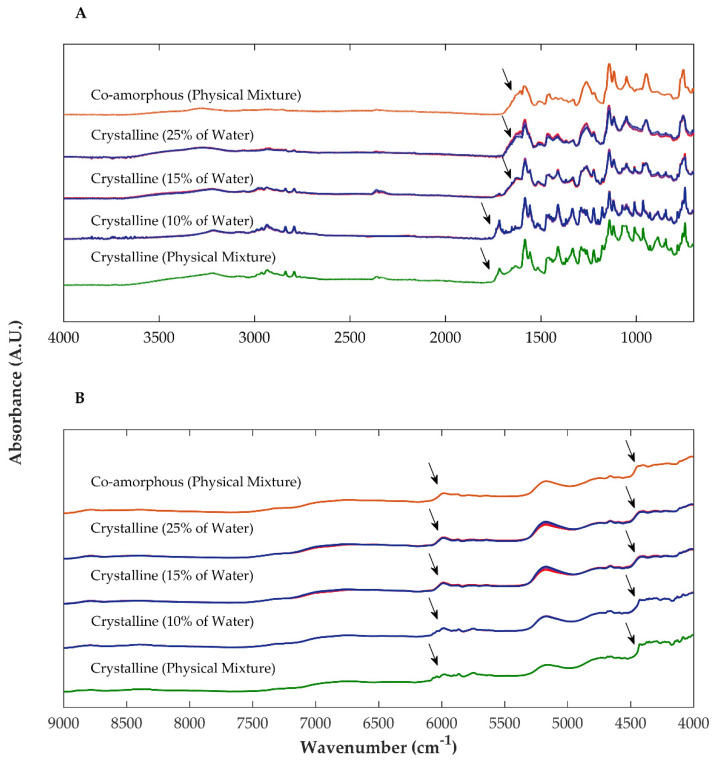
Fourier-transformed mid (**A**) and near (**B**) infrared spectra of formulations containing crystalline olanzapine and saccharin as physical mixtures (green) and after granulation with different fractions of water (10, 15 and 25%). For comparison purposes, the spectra of co-amorphous olanzapine and saccharin in a physical mixture (orange) were plotted. Drying temperature—40 °C (dark blue), 65 °C (purple) and 90 °C (red).

Regarding the formulation containing crystalline OLZ and SAC (formulation B), significant variations in the FTIR and NIR spectra were observed for samples collected throughout the process, compared to the sample obtained by mixing the compounds. These variations were particularly relevant when the fraction of water added to the powder blend was increased from 10 to 25% (Figure 7). Spectra of samples granulated with 10% of water presented peaks in FTIR and NIR spectra at the same wavenumbers and with similar intensities compared to those of the physical mixture. When the fraction of water was increased to 25 or 40%, the spectral changes suggest the formation of a salt between OLZ and SAC, as discussed before, and support the co-amorphization of both substances. This observation is supported by the fact that the spectra of these samples were similar to those of the physical mixture containing the OLZ-CAM produced by solvent evaporation. 

In order to identify the threshold, i.e., the amount of water required for wetting the physical mixture to present the same spectrum as the dry blend, additional experiments were performed using water contents between 5 and 15% of water (*w*/*w*, dry basis). Samples of wet masses with 5 and 10% (*w*/*w*) water produced spectra with identical features to the ones observed for the dry blend. On the contrary, spectra similar to those of blends containing OLZ-CAM were obtained for samples taken from wet masses with 15% (*w*/*w*) water. This suggests that wet masses obtained from physical mixtures containing OLZ and SAC generated OLZ-CAM, when a minimal fraction of water (15% *w*/*w*) was used (Figure 7). 

**Figure 8:**
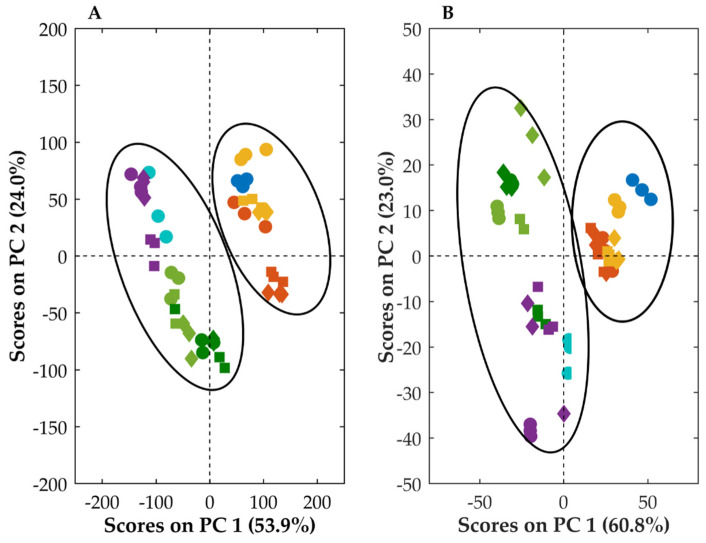
Principal component analysis conducted on the Fourier-transformed mid (**A**) and near (**B**) infrared spectral data of formulations containing crystalline olanzapine and saccharin in a physical mixture (dark blue) and in granules (5, yellow, 10, orange, 15, dark green and 25%, light green, of water) and to formulations containing co-amorphous olanzapine and saccharin, after physical mixture (light blue) and granulation using 10% of water (purple). Drying temperature applied to samples: 40 °C (circle), 65 °C (square) and 90 °C (diamond).

To further analyze and compare the spectra of samples containing OLZ and SAC in the crystalline and amorphous forms, a principal component analysis was conducted. The analysis has allowed the construction of models for both FTIR and NIR data, describing approximately 96% of the variation present in the dataset, reflecting the fitting of the principal component models to the data. The formation of clusters has confirmed the similarity between the spectra of materials present in samples [59,60]. In this respect, the models have shown that blends granulated with 5 or 10% of water were in the same cluster as samples collected from the physical mixture containing crystalline OLZ and SAC (Figure 8). In contrast, the granulation of the physical mixture with 15 and 25% of water resulted in samples in which a deviation in the score plot from the aforementioned cluster was observed. This was used to confirm the differences between the spectra of these samples and thus to infer the variation in the intermolecular interactions established between OLZ and SAC molecules. Additionally, the models have shown that samples granulated with 15 and 25% of water were in the same cluster of samples as the physical mixture containing the CAM produced by solvent evaporation prior to the production of granules. 

Overall, the spectroscopic models demonstrated that the granulation of blends containing OLZ and SAC (with 15 or 25% of water) enabled the in situ formation of the CAM (based on the similarity of FTIR and NIR spectra). Co-amorphization is thus likely to occur during a traditional, inexpensive granulation process. 

#### 2.2.3. Dissolution Tests

Amorphization and co-amorphization of drugs are valuable strategies used to enhance drugs’ solubility and dissolution rate and, subsequently, dissolution tests are paramount to confirm the advantages of these materials. Prior to the dissolution tests, samples were gently milled to ensure particle size homogeneity (125–180 µm) and minimize the impact of particle size on the dissolution profile. 

**Figure 9:**
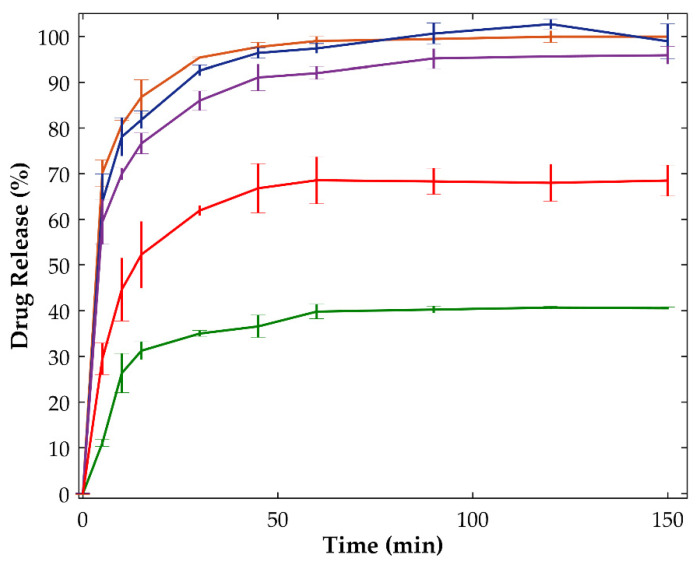
Dissolution profiles of samples containing the physical mixture of crystalline olanzapine (green), the physical mixture of quench cooled olanzapine (orange) and granules (25% of water) containing quench cooled olanzapine dried at 40 °C (blue), 65 °C (purple) and 90 °C (red) (n = 3, 100 rpm, non-sink conditions, 500 mL, 37 °C, phosphate buffer pH 8.0).

At the end of the dissolution tests, samples made of crystalline OLZ (as raw material, formulation A) released about 41% of the drug (equivalent to 37 mg/L), regardless of the processing conditions applied (Figure 9). Moreover, the amorphization of OLZ enabled the dissolution of the total amount of the drug present in the dissolution vessel (equivalent to 90 mg/L), at a higher dissolution rate. In fact, the time required to release 80% of the equilibrium concentration was significantly reduced as a result of the amorphization of the drug, from 20 min (physical mixture containing the crystalline drug) to 10 min (physical mixture containing the amorphous drug). These results reinforce the advantages of the conversion of the crystalline into amorphous forms and may anticipate a higher bioavailability for the latter.

As shown previously, the processing of amorphous OLZ during the manufacture of granules, extrudates and pellets resulted in a significant increase in the degree of recrystallization of the drug. The highest extent of recrystallization was observed when granules were dried at the highest temperature (90 °C, with 25% of water), resulting in a significant deviation of the dissolution profile compared to those profiles of samples of granules dried at lower temperatures (Figure 9). For granules dried at 90 °C (25% of water), the recrystallization of the drug resulted in a dissolution profile markedly different from those of granules dried at lower temperatures (Figure 9) and reduced drug release (68%). The differences in the equilibrium concentration observed for granules dried at 90 °C, compared to the physical mixture containing crystalline OLZ, were most likely related to residual traces of amorphous material in granulated samples. Furthermore, granules dried at 40 and 65 °C showed a higher extent of release of the drug (98.9 ± 3.8 and 95.9 ± 1.9%, respectively). 

For further evaluation, dissolution profiles were compared using the model-independent similarity factor (f_2_). Compared to the dissolution profile of amorphous OLZ (physical mixture, formulation A), a f_2_ of approximately 76 and 53 was obtained for granules dried at 40 and 65 °C, respectively. According to the FDA [61], a f_2_ > 50 confirms the similarity between dissolution profiles. In this respect, the values obtained suggested that the granules dried at 40 and 65 °C presented a drug release comparable to that of the physical mixture produced with quench cooled OLZ.

**Figure 10:**
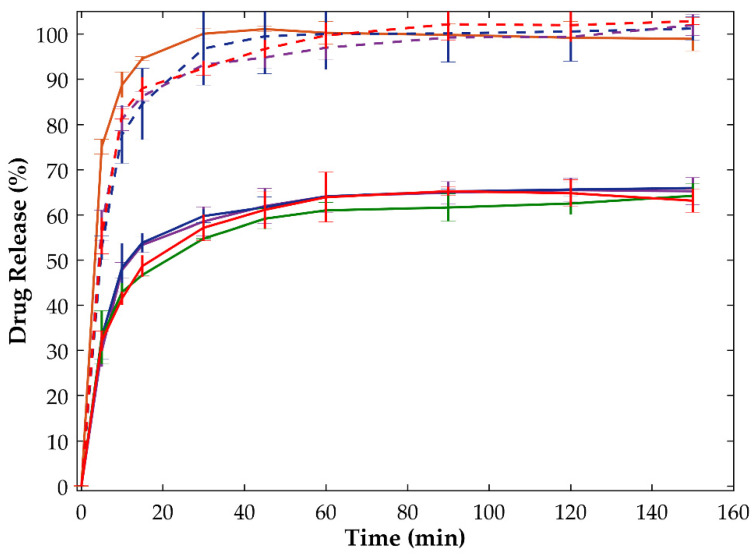
Dissolution tests conducted with crystalline olanzapine:saccharin (physical mixture, green solid line), co-amorphous olanzapine:saccharin (physical mixture, orange solid line) and granulated olanzapine:saccharin formulation using 10% (solid line) and 25% of water (dashed line). Drying temperature applied to samples: 40 °C (blue), 65 °C (purple) and 90 °C (red).

When crystalline OLZ was formulated together with SAC (formulation B), a slight increase in the equilibrium concentration of the drug (at approximately 56.7 mg/L, equivalent to a drug release of 63%) was observed. Granulation of the powder mixture with 5% or 10% (Figure 10) of water produced granules which released the drug in similar concentrations at equilibrium and presented similar dissolution rates compared to those of the respective physical mixture. In contrast, the in situ co-amorphization of OLZ, achieved by the addition of high fractions of water to the physical mixture containing crystalline OLZ and SAC (25% of water, as an example (Figure 10)), enhanced both the dissolution rate and the concentration of the drug at equilibrium. Interestingly, samples from granules in which 25 or 40% of water was used have shown identical dissolution profiles compared to those of the formulation containing the CAM prepared by solvent evaporation prior to the production of granules and extrudates (Figure 10). In this regard, samples granulated with 25 or 40% of water took about 10 to 15 min to release 80% of the drug, whereas granules made of crystalline OLZ in a physical mixture required approximately 21 min to release 50% of the drug (80% of the equilibrium concentration). The lower release rate of OLZ from granules made with 25 or 40% of water, compared to the ones containing the OLZ-CAM (<10 min to release 80% of OLZ), may be justified by the presence of crystalline nuclei in the granules after drying, emphasizing the advantages of CAMs prepared a priori or in situ, based on the extent and rate of dissolution, as compared to the crystalline counterpart. 

## 3. Materials and Methods

### 3.1. Materials

OLZ (polymorphic form I, melting point 192.4 °C [35]) was used as a model drug (Rampex Labs Pvt. Ltd., Telangana, India). For the preparation of amorphous OLZ, liquid nitrogen (purity ≥ 99.8%, Air Liquide, Lisbon, Portugal) was used to rapidly quench the molten drug. For the preparation of OLZ-CAM, SAC was used as co-former (Sigma-Aldrich, Steinheim, Germany) and dichloromethane was used as solvent (Biochem Chemopharma, Cosne sur Loire, France). To produce granules and pellets, OLZ was incorporated in a formulation made of dibasic calcium phosphate anhydrous (DI-CAFOS^®^ A60, Budenheim, Budenheim, Germany), microcrystalline cellulose (Avicel PH-101, FMC Corp., Cork, Ireland) and polyvinylpyrrolidone (K25, BASF, Ludwigshafen, Germany). Demineralized water, used as granulation liquid, was obtained using a Destillo 2 apparatus (Herco, Freiberg am Neckar, Germany). Hard gelatin capsules (size 0) were acquired from Lonza (Basel, Switzerland). To prepare the pH 8.0 phosphate buffer for the dissolution studies, demineralized water (Destillo 2 apparatus, Herco, Freiberg am Neckar, Germany), sodium hydroxide (Eka Chemicals Inc., Marietta, GA, USA) and potassium phosphate monobasic (Carlo Erba Reagents, Val de Reuil, France) were used. 

### 3.2. Methods

#### 3.2.1. Preparation of Amorphous and Co-Amorphous Olanzapine

Amorphization and co-amorphization of OLZ were achieved using the quench cooling and solvent evaporation procedures, respectively, which delivered the highest fractions of amorphous contents for each species [35]. 

Preparation of amorphous OLZ: Amorphization of OLZ was achieved by quench cooling. In brief, the drug was heated up to 200 °C to assure complete melting and then liquid nitrogen was rapidly poured onto the molten drug to preserve its molecular conformation.

Preparation of co-amorphous OLZ: A mixture made of OLZ and SAC, in a 1:1 molar ratio, was dissolved in pure dichloromethane prior to evaporation of the solvent under vacuum conditions (45 °C, 650 mbar, R-100, Buchi Rotavapor, Flawil, Switzerland). The product obtained was left under vacuum for 24 h to ensure the total removal of the solvent, as described elsewhere [35]. 

#### 3.2.2. Characterization of Amorphous and Co-Amorphous Olanzapine

Amorphous OLZ and OLZ-CAM were characterized, immediately after preparation, using DSC and XRPD to confirm the preparation of amorphous entities. Prior to characterization, amorphous OLZ and OLZ-CAM were gently milled, using a mortar and pestle, and passed through a 180 µm mesh sieve to ensure particle size uniformity (125–180 µm fraction). 

X-ray powder diffraction: XRPD measurements were conducted using a PANalytical X-ray diffractometer (X’Pert PRO, PANalytical, Almelo, The Netherlands). A CuKα source of radiation (λ = 1.54 Å) at 40 kV and 30 mA was applied to samples at a step size of 0.017 °2θ, a counting time of 19.685 s (5.20 °2θ/min) and considering the analysis range of 7–35 °2θ.

Differential scanning calorimetry: Thermal analysis of crystalline and amorphous materials was performed using a calorimeter (Discovery 25, TA Instruments, New Castle, DE, USA). Prior to analysis, a sample (ca. 7.5 mg) was carefully weighted and sealed in aluminum pans (THEPRO GbR, Heinsberg, Germany). The calorimeter was operated in a modulated fashion within the temperature interval −65 to 250 °C, at a predefined heating rate of 5 °C/min, at an amplitude of 0.796 °C, for a period of 60 s, using nitrogen as purging gas (50 mL/min). The thermograms were analyzed using the TRIOS software (TRIOS, version 5.1.1, 2020, TA Instruments, New Castle, USA). The T_g_ of amorphous and OLZ-CAM was calculated as the midpoint of the change in the heat flow baseline. The enthalpy of recrystallization was determined as the area of the exothermic event observed in the non-reversing heat flow curve. 

Fourier-transformed mid infrared spectroscopy: FTIR spectroscopy measurements were performed on a Bruker spectrometer (Alpha II, Bruker, Billerica, MA, USA) connected to a diamond ATR accessory (Platinum ATR, Bruker, Billerica, MA, USA). Powdered samples (n = 3) were scanned 24 times in the wavenumber interval 4000–700 cm^−1^ at a resolution of 4 cm^−1^. The spectra acquired were analyzed and results treated using the Spectragryph software (version 1.2.13, 2019, Oberstdorf, Germany). 

Near infrared spectroscopy: NIR spectra were acquired using a TLA spectrophotometer (TLA 2000, ABB, Québec, Canada) equipped with an indium-gallium-arsenide detector and using polytetrafluoroethylene (PTFE) as background (SKG8613G, ABB, Montreal, QC, Canada). Powdered samples (n = 3) were scanned 32 times in the wavenumber interval of 10,000–4000 cm^−1^, using a resolution of 8 cm^−1^. The spectra acquired were analyzed using the Spectragryph software (version 1.2.13, 2019, Oberstdorf, Germany).

#### 3.2.3. Formulation

After ensuring the amorphization or co-amorphization of OLZ, amorphous and OLZ-CAM were incorporated in formulations intended for the manufacture of granules, extrudates and pellets. The formulations considered excipients commonly used by the pharmaceutical industry, such as dibasic calcium phosphate anhydrous, microcrystalline cellulose or polyvinylpyrrolidone (Table 2). Furthermore, the selection of these formulations was made taking into consideration the versatility of these powder blends for the production of different oral solid dosage forms including tablets, pellets and granules. Both formulations (A and B) included OLZ (at a constant fraction of 30%) with formulation A containing either crystalline or amorphous OLZ (without SAC), whereas formulation B contained either the physical mixture of crystalline OLZ and SAC or the CAM obtained by solvent evaporation (Table 2).

**Table 2:** Composition (%, *w*/*w*) of formulation A and formulation B.

Component	Formulation	
	A	B
Olanzapine	30	30
Saccharin	0	18
Dibasic calcium phosphate anhydrous	45	27
Microcrystalline cellulose	20	20
Polyvinylpyrrolidone	5	5

#### 3.2.4. Preparation of Granules, Extrudates and Pellets

Mixtures (30 g of either formulation A or B; Table 2) were blended in a planetary mixer (Chef, Kenwood, New Lane, United Kingdom) for 10 min prior to the addition of the granulation liquid (demineralized water) at 10, 25 or 40% (*w*/*w*) of the dried powder. Samples of the physical mixture of all components, except water, were used as controls for comparison purposes. Wet masses were blended for an additional 10 min prior to drying to constant weight in ovens (UM 100, Memmert, Schwabach, Germany) set to different temperatures (40, 65 or 90 °C). 

To produce extrudates and pellets, 30 g of the wet masses (containing 40 or 10% *w*/*w* of water for formulation A and B, respectively) were stored in polyethylene bags for 24 h at room temperature (23 °C) prior to extrusion and spheronization. Prior to extrusion, a fraction of the wet masses, stored for 24 h, was collected and dried (40, 65 or 90 °C) to evaluate whether OLZ had undergone solid-state modifications. Extrusion was carried out to the wet masses in a universal testing machine (Lloyd Instruments, LR50K Plus, Largo, FL, USA) equipped with a 50 kN load cell. A die with a length-to-diameter ratio of 4 and a test speed of 200 mm/min was considered to manufacture extrudates. The extrudates were spheronized in a radial plate spheronizer (230, Caleva, Dorset, UK) at 1000 rpm, for 20 min, and the pellets obtained dried to constant weight in an oven, set to 40 °C (UM 100, Memmert, Schwabach, Germany).

Finally, granules, extrudates and pellets were gently milled with a mortar and pestle and passed through a 180 µm mesh sieve to guarantee particle size homogeneity (125–180 µm fraction). Fractions of about 66.5 mg (equivalent to 20 mg of OLZ) were weighted and introduced manually in hard gelatin capsules (size 0); capsules prepared for the dissolution testing contained 150 mg of pellets (equivalent to 45 mg of OLZ).

#### 3.2.5. Characterization of Granules, Extrudates and Pellets

Characterization of the physical mixtures, granules, extrudates and pellets (n = 3) was conducted 1 day after preparation. Masses, characterized prior to granulation (low shear) or extrusion (high shear), were used as controls to eliminate the effect of stress induced by the unit operations.

Assessment of the solid-state modifications of OLZ during processing: Samples of the different materials were characterized using DSC, XRPD, FTIR and NIR as described previously (see Section 3.2.2). Analysis of the results was conducted using the Modde Pro software (version 13.0.1.27179, 2021, Gottingen, Germany).

Dissolution studies: Dissolution tests were carried out in a Sotax dissolution apparatus (AT7, Sotax, Aesch, Switzerland) on capsules containing OLZ (formulations A or B) using the paddle method (500 mL of pH 8.0 phosphate buffer—equivalent to 90 mg/L of OLZ, i.e., non-sink conditions—as dissolution medium, 37 ± 0.5 °C, 100 rpm). Samples (2 mL) of dissolution media were withdrawn at 5, 10, 15, 30, 45, 60, 90, 120 and 150 min and replaced by fresh dissolution medium to maintain the dissolution volume constant throughout the duration of the test. Non-sink conditions for crystalline OLZ were maintained throughout the test, as reflected by the plateau reaching well below the quantity of OLZ in the vessel. The dissolution samples were passed through a 0.22 µm MCE filter (Merck, Boston, MA, USA) and analyzed using UV spectrophotometry (λ = 254 nm, U-1900, Hitachi, Tokyo, Japan) to determine the fraction of OLZ released at each time. 

#### 3.2.6. Statistical Analysis

Results were analyzed using one-way ANOVA (SPSS Statistics, version 27.0.1.0, 2020, IBM, New York, NY, USA) and when statistical significance (*p* < 0.05) was observed, the post hoc Tukey’s test conducted for comparison purposes. 

#### 3.2.7. Principal Component Analysis

Principal component analysis was carried out on the entire FTIR and NIR spectra using the Matlab software (R2015a, 2015, MathWorks, Sherborn, MA, USA). Prior to analysis, the FTIR and NIR spectra were pre-treated with the multiplicative scatter correction to remove undesirable scatter effects and to compensate additive and/or multiplicative effects in the spectra [62,63].

## 4. Conclusions

The work demonstrated that the co-amorphization of OLZ and SAC as a co-former resulted in a powder with appropriate stability to the stress conditions considered in the study, since no loss of amorphous content was observed. On the contrary, pure amorphous OLZ showed significant recrystallization, as a result of exposure to water (wetting of the masses) and heat (drying temperature). The addition of high fractions of water and high drying temperatures to the powdered system containing amorphous OLZ resulted in granules containing OLZ, predominantly in its crystalline form. In this respect, the selection of low drying temperatures and utilization of low fractions of the granulation liquid should be emphasized to minimize the recrystallization of the amorphous fraction of drugs. Interestingly, the addition of high fractions of water to the physical mixture composed of crystalline OLZ and SAC promoted the co-amorphization of the drug and co-former, as suggested by the XRPD, spectroscopic analysis (FTIR and NIR) and dissolution studies. The in situ co-amorphization of OLZ during granulation avoids the preparation of the CAM prior to the process, thus reducing the processing costs and stability issues. In summary, this study has provided new insights regarding the advantages of CAMs for the production of more bioavailable solid dosage forms in view of the higher solubility and dissolution rate. Further studies are needed to evaluate whether the in situ co-amorphization is also observed when other drugs, or co-formers, are considered.

## Figures and Tables

**Table 1:** Area under the peak (intensity.°2θ, 20.7–21.2°2θ) and enthalpy of recrystallization (J/g) of olanzapine present in physical mixtures (either crystalline or quench cooled olanzapine) and samples after processing.

Sample		Drying Temperature (°C)	Area under the Peak (Intensity.°2θ)	Enthalpy of Recrystallization (J/g)
**OLZ QC** (**amorphous**)	Physical Mixture		0.079 ± 0.002 ^##^	12.61 ± 0.11 ^##^
	Granules (10% water)	40	0.079 ± 0.002 ^##^	11.80 ± 0.61 ^##^
		65	0.084 ± 0.003 ^##^	10.82 ± 0.31 *^,##^
		90	0.099 ± 0.003 **^,#^	7.78 ± 0.88 **^,##^
	Granules (25% water)	40	0.081 ± 0.001 ^##^	10.91 ± 0.53 *^,##^
		65	0.095 ± 0.002 **^,#^	9.40 ± 0.25 **^,##^
		90	0.102 ± 0.002 **	5.09 ± 0.63 **^,##^
	Granules (40% water)	40	0.091 ± 0.003 *^,##^	8.49 ± 0.09 **^,##^
		65	0.101 ± 0.002 **	5.62 ± 0.20 **^,##^
		90	0.107 ± 0.001 **	4.16 ± 0.10 **^,##^
	Wet Masses (24 h storage) (40% water)	40	0.105 ± 0.001 **	3.63 ± 0.20 **^,##^
		65	0.104 ± 0.003 **	2.15 ± 0.25 **^,#^
		90	0.107 ± 0.001 **	2.00 ± 0.20 **^,#^
	Extrudate	40	0.107 ± 0.002 **	1.26 ± 0.21 **
	Pellet	40	0.108 ± 0.001 **	0.00 ± 0.00 ^(a),^**
**OLZ RM** **(crystalline)**	Physical Mixture		0.107 ± 0.003 **	0.00 ± 0.00 ^(a),^**

OLZ, Olanzapine; QC, Quench cooled; RM, Raw material; ^(a)^ Not detected; * *p* < 0.05 and ** *p* < 0.01 versus the physical mixture containing quench cooled OLZ; ^#^ *p* < 0.05 and ^##^ *p* < 0.01 versus the physical mixture containing crystalline OLZ.

## Data Availability

Not applicable; the data presented is not publicly available.

## Supplementary Materials

The supporting information can be downloaded at: https://www.mdpi.com/article/10.3390/ijms231810234/s1.
