# Supplementary material for: Amorphous and Co-Amorphous Olanzapine Stability in Formulations Intended for Wet Granulation and Pelletization
Source: Int J Mol Sci. 2022 Sep 6;23(18):10234. doi: 10.3390/ijms231810234 (PMC9499418; doi:10.3390/ijms231810234)
Supplement: Supplementary file 1 [file ijms-23-10234-s001.zip › ijms-1863691-supplementary.pdf]

CAPTIONS TO SUPPLEMENTARY FIGURES

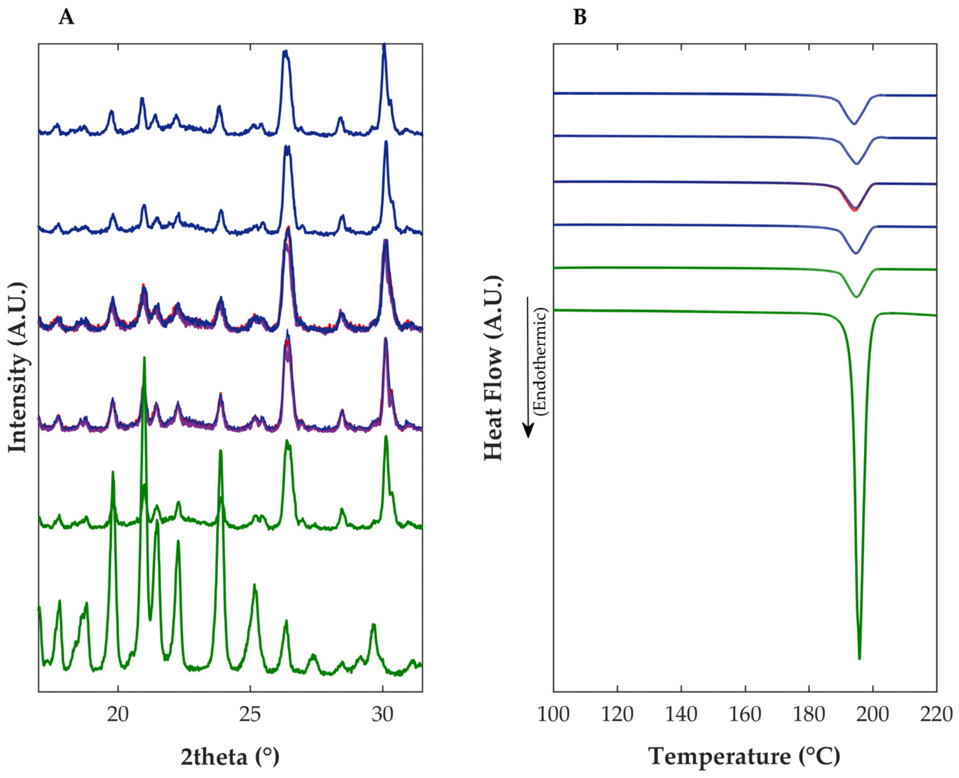

Figure S1. X-ray powder diffractograms (A) and differential scanning calorimetry thermograms (B) of pure crystalline olanzapine and samples containing crystalline olanzapine (formulation A) after physical mixture, incorporation of 40% of water, storage for 24 h, extrusion and spheronization (from bottom to the top) [coloration according to the drying temperature applied to samples: 40°C (dark blue), 65°C (purple) and 90°C (red)].

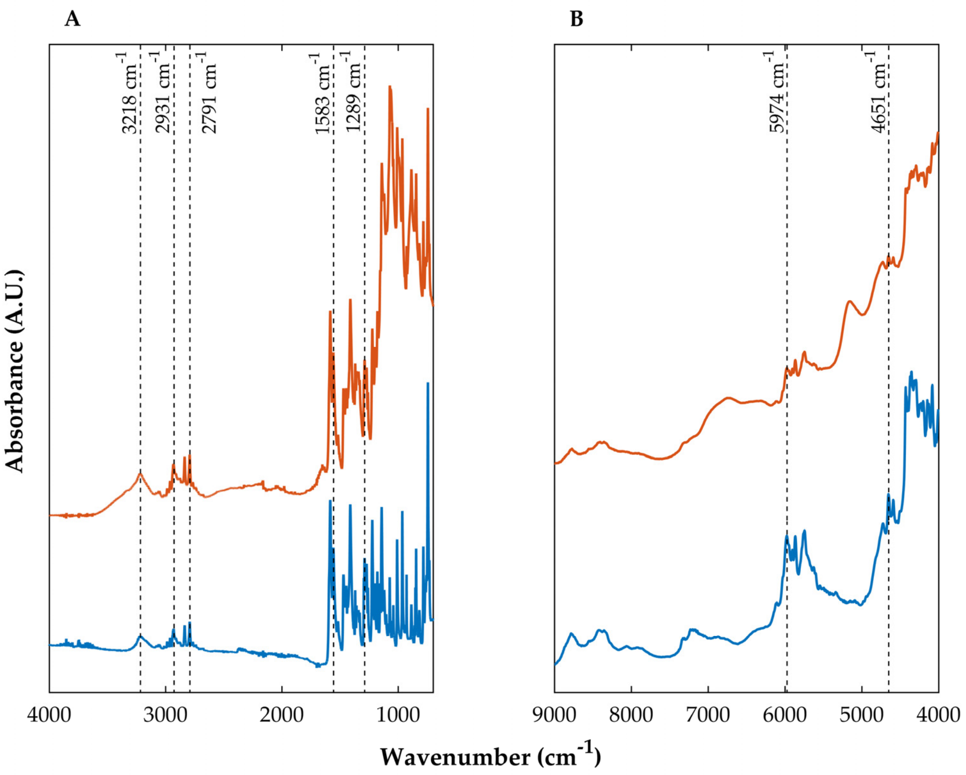

Figure S2. Fourier-transform mid (A) and near infrared spectra (B) of pure crystalline olanzapine (in blue) and after blending with the excipients described in Table 2 (formulation A, in orange).

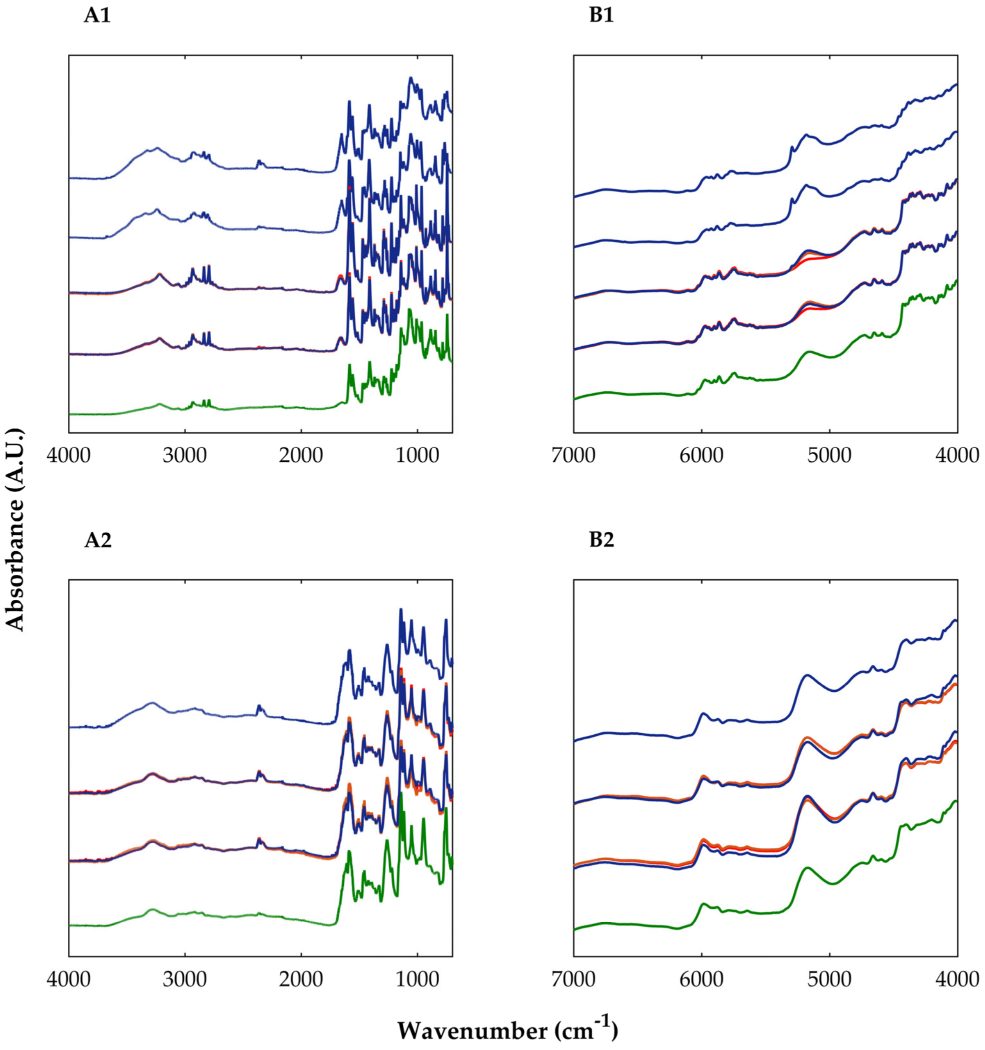

Figure S3. Fourier-transform mid (A) and near infrared spectra (B) of formulations containing crystalline olanzapine (1, formulation A) or co-amorphous olanzapine (2, formulation B) after processing [from bottom to the top: after physical mixture, granulation, storage for 24 h, extrusion and spheronization (if applicable), coloration according to the drying temperature applied to samples: 40°C (dark blue), 65°C (purple) and 90°C (red)].
